# Non-invasive ventilation for the management of children with bronchiolitis (NOVEMBR): a feasibility study and core outcome set development protocol

**DOI:** 10.1186/s13063-018-2969-9

**Published:** 2018-11-14

**Authors:** Clare van Miert, Ricardo M. Fernandes, Helen Eccleson, Emma Bedson, Steven Lane, Matthew Peak, Kent Thorburn, Vanessa Compton, Kerry Woolfall, David Lacy, Paula Williamson, Paul S. McNamara

**Affiliations:** 1 School of Nursing and Allied Health, Liverpool John Moores University, Room 3.12 Henry Cotton Building, 15-21 Webster Street, Liverpool, L3 2ET UK; 2 Paediatric Medicines Research Unit, Alder Hey Children’s NHS Foundation Hospital Trust, Eaton Road, Liverpool, L12 2AP UK; 3 Clinical Pharmacology Lab/Unit, Faculty of Medicine and Instituto de Medicina Molecular, Universidade de Lisboa, Lisbon, Portugal; 4 Department of Pediatrics, Hospital Santa Maria, Lisbon, Portugal; 5 Medicines for Children Clinical Trials Unit, Clinical Trials Research Centre, University of Liverpool, Institute of Child Health, Alder Hey Children’s NHS Foundation Trust, Liverpool, L12 2AP UK; 6 Department of Biostatistics, Block F, Waterhouse Building, 1-5 Brownlow Street, Liverpool, L69 3GL UK; 7 Paediatric Intensive Care Unit, Alder Hey Children’s NHS Foundation Hospital Trust, Eaton Road, Liverpool, L12 2AP UK; 8 MRC North West Hub for Trials Methodology Research Institute of Psychology, Health and Society, Block B, Room B112, 1st Floor Waterhouse Building, Liverpool, L69 3GL UK; 9 Department of Paediatrics, Wirral University Teaching Hospital NHS Foundation Trust, Arrowe Park Road, Upton, Wirral, CH49 5PE UK; 10 Institute of Child Health, University of Liverpool, Alder Hey Children’s NHS Foundation Trust, Eaton Road, Liverpool, L12 2AP UK

**Keywords:** Bronchiolitis, High-flow nasal cannula, Nasal continuous positive airway pressure, Oxygen inhalation therapy, Core outcome sets

## Abstract

**Background:**

Bronchiolitis is an acute lower respiratory infection which predominantly affects young children. Treatment for bronchiolitis is limited to supportive therapy. Nasal oxygen therapy is part of routine care, and delivery now incorporates varying levels of non-invasive continuous positive airway pressure and/or high-flow nasal cannula oxygen therapy. Despite wide clinical use, there remains a lack of evidence on the comparative effectiveness and safety of these interventions. Furthermore, research in this field is hampered by the use of multiple outcome measures in current clinical trials.

**Methods/design:**

This mixed methods study includes a systematic review of outcome measures, telephone interviews with parents, focus group workshops and a Delphi survey with healthcare professionals and parents. These methods will be used to identify and prioritise outcomes for inclusion in a core outcome set and to explore issues pertinent to the design of a future randomised controlled trial comparing different modes of oxygen therapy for bronchiolitis. UK hospitals will also be contacted and asked to complete a survey to provide an overview of current practice to enable assessment of capability and capacity to run a future clinical trial.

**Discussion:**

This study will facilitate the design of a future clinical trial of non-invasive ventilation in children with bronchiolitis which is acceptable to important stakeholders. Furthermore, core outcome set development will improve standardisation, measurement and reporting of clinically important outcomes in bronchiolitis.

**Trial registration:**

ISRCTN Registry, ISRCTN75766048. Registered on 18 December 2017. This study was retrospectively registered in the ISRCTN Registry and on the Core Outcome Measures in Effectiveness Trials (COMET) Initiative database (15 September 2017).

**Electronic supplementary material:**

The online version of this article (10.1186/s13063-018-2969-9) contains supplementary material, which is available to authorized users.

## Background

Bronchiolitis is an acute viral lower respiratory tract infection which predominantly affects children up to two years of age. Respiratory syncytial virus (RSV) is the most common cause of bronchiolitis [1]. Symptoms comprise a coryzal prodrome lasting approximately three days, persistent cough, increased respiratory rate, chest recession and wheeze or crackles on auscultation [2]. Most children with bronchiolitis have mild symptoms and can be managed at home [3]. However, up to 3% of all infants are hospitalised with this condition, most commonly between 1 and 6 months of age [4, 5]. Between 2004 and 2011, admissions for bronchiolitis in the UK rose by 60% [6]. The median duration for hospitalisation with this condition varies substantially across the UK and Europe [7], likely reflecting variations in clinical practice and socio-economic conditions [4, 8]. A significant proportion of infants present to healthcare services with severe disease or deteriorate whilst in hospital, requiring intensive care [9, 10].

Treatment for bronchiolitis is principally supportive, comprising oxygen and fluids/nutritional support [2]. A number of treatment interventions have been assessed in clinical trials and systematic reviews, including antibiotics, bronchodilators, chest physiotherapy, epinephrine, leukotriene inhibitors, glucocorticoids, heliox, hypertonic saline, immunoglobulin, inhaled corticosteroids, oxygen therapy, recombinant deoxyribonuclease (rhDNase), steam inhalation and humidity, exogenous surfactant (in mechanically ventilated infants), nasal continuous positive airway pressure (nCPAP) and high-flow nasal cannula (HFNC) [11–24]. Oxygen therapy and the use of oximetry is the only intervention that has had a major impact on survival over the last 40 years, contributing to a reduction in mortality rates from around 20% to < 1% [1, 25]. Over the last two decades, giving oxygen nasally and non-invasively with varying levels of positive airway pressure or flow has become an important part of the routine clinical management of hospitalised children with bronchiolitis, particularly those with severe disease [26–28]. This is done in one of two ways, with nCPAP or HFNC [29].

### Nasal continuous positive airway pressure (nCPAP)

This method works by delivering an air/oxygen mixture at a preset distending airway pressure, thereby widening peripheral airways and allowing deflation of over-distended lungs [30]. It is widely used to treat respiratory distress and reduce the need for intubation and invasive ventilation in infants with worsening bronchiolitis, particularly in critical care settings [31]. In spite of its widespread use, there is little good-quality research on the efficacy of nCPAP or optimum thresholds for its initiation. Observational studies provide some indication that nCPAP may provide an alternative to mechanical ventilation, based on improvement in physiological parameters and temporal trends showing reduction in invasive ventilation in bronchiolitis [27–29]. A Cochrane systematic review published in January 2015 [21] found only two small, randomised controlled trials (RCTs) [32, 33] that showed improvement in their respective primary outcomes: partial pressure of carbon dioxide (PCO_2_) reduction; respiratory distress score. However, both RCTs had major methodological flaws, including small sample sizes, short protocol durations and the use of surrogate endpoints of questionable relevance either clinically or to patients/families. The Cochrane ‘bottom line’ was that the effect of nCPAP in children with acute bronchiolitis is uncertain and larger trials with adequate power are needed [21].

### High-flow nasal cannula (HFNC)

HFNC is a recent introduction to clinical practice. The precise mechanism of action is unclear but thought to support effort of breathing through providing a distending pressure [29]. Furthermore, it allows humidified warm high-flow air/oxygen blends to be delivered using specific cannulas and has potential for use in different settings, including critical care, high-dependency units, general wards and emergency departments (EDs) [30]. Proof-of-concept studies focusing on physiologic outcomes have shown promise, and observational evidence also suggests clinical benefit from the use of HFNC in bronchiolitis [34–36]. However, a Cochrane review published in 2014 found only one pilot clinical trial of sufficient quality for inclusion and concluded that further research needed to be undertaken [37]. More recently, the first published RCT comparing HFNC with low-flow oxygen (2 L/min nasal cannula wall oxygen) in inpatients with bronchiolitis suggested a benefit in reducing treatment failure, despite no change in duration of oxygen therapy [38].

### nCPAP vs HFNC

Compared to nCPAP, it is believed that HFNC is better tolerated and more straightforward to use, may reduce skin and nasal trauma and is associated with reduced costs [39–42]. Whilst further studies are currently underway to clarify the benefits and risks of HFNC compared to low-flow oxygen, the first head-to-head comparisons between nCPAP and HFNC have been recently published [39, 43]. The TRAMONTANE multi-centred RCT, conducted in paediatric intensive care units in France, suggested that respiratory support provision by nCPAP may be more efficient than that by HFNC [39]. However, many gaps in evidence remain regarding the comparative efficacy and safety of both treatments for different levels of bronchiolitis severity. Future RCTs should consider feasibility across settings, thresholds of use, and adequate outcome selection.

### Outcome selection for use in clinical trials of bronchiolitis

Selection of appropriate primary and secondary outcomes is essential for study design, as ultimately any study is only as credible as its endpoints [44]. To be useful, clinical trials that evaluate benefits and harms of interventions must select outcomes of relevance to stakeholders and measure them using instruments with adequate measurement properties [45]. Inconsistent selection, measurement and reporting of outcomes in clinical trials raise three problems [46]. First, outcomes may not consistently reflect endpoints that are meaningful for all stakeholders, particularly parents and caregivers or healthcare professionals (HCPs) in different settings. Second, inconsistency in measurement domains and instruments is a barrier to compare, contrast and combine trial findings, which will inevitably affect their interpretation and future uptake. Third, if researchers have measured a particular outcome in a variety of ways, outcome reporting bias may ensue.

These issues could be addressed with the development and application of an agreed standardised set of outcomes (a ‘core outcome set’) pertinent to all stakeholders, especially patients/carers and HCPs [46, 47]. To develop a core outcome set (COS), one must distinguish between potential domains (’what to measure’) and measurement instruments (’how to measure’) and define the process to identify them and to reach consensus on which to include in a COS. Considerable methodological evidence has accumulated in this field, and guidance is available to support the development, implementation, evaluation and updating of a COS [48]. The recent handbook by the COMET Initiative recommends a four-step process to develop a COS [48].

One of the key limitations identified by most systematic reviews of treatments in acute viral bronchiolitis across different settings has been the heterogeneity in the selection of outcomes and measurements reported in clinical trials [49]. However, the extent of this heterogeneity is unknown. A recent nationwide online survey conducted in Portugal identified outcomes of relevance to paediatricians and general practitioners [50] but did not seek the perspectives of parents or other stakeholders, and no consensus procedure was undertaken. No COS has been previously developed for this condition.

## Methods/design

### Aims and objectives

The long-term aim of the NOVEMBR study is to find out how to best provide respiratory support to children with bronchiolitis when they are admitted to hospital (see Additional file 1).

The specific aims of the NOVEMBR feasibility study are to:

1. Develop a COS for use in future clinical trials in bronchiolitis
2. Explore issues critical to the design of an RCT of non-invasive ventilation in children with bronchiolitis
3. Comprehensively assess current UK practice as regards bronchiolitis management, potential trial capability and acceptability

### Setting

This multi-centre study will be undertaken at seven UK National Health Service (NHS) hospitals (district general hospitals [*n* = 4] and paediatric tertiary centres [*n* = 3]).

### Stakeholder involvement

An important aspect of the NOVEMBR study is consultation with and involvement of key stakeholders (parents/carers and HCPs) at all stages. The purpose of involving these parties is to ensure capture of their unique experiences and opinions. This knowledge will inform the design of a future RCT and COS development in the broadest sense.

Two parents were consulted in the initial stages of the design of the study. They discussed their experiences of having a child admitted to hospital with bronchiolitis and the treatment received. Discussion with these parents highlighted a number of fundamental issues for exploration within the proposed study, including:

- Parental capacity to provide informed consent in the emergency setting
- Acceptability of deferred consent
- Route and time from admission to diagnosis and intervention (e.g. starting nCPAP or HFNC)
- Infant’s intolerance of nCPAP
- Parental priorities in consent decision-making
- Concerns about the long-term impact of bronchiolitis
- Willingness to participate in any RCT, including follow-up stages

Parent participation in the study was discussed. Both parents said they thought parents would appreciate the opportunity to meet other parents with similar experiences and discuss the RCT in a workshop event. Furthermore, both parents recommended that workshop events and interviews should be incorporated into the NOVEMBR feasibility study protocol.

### Study management and oversight

The study will be overseen by a study management group (SMG) and a Study Steering Committee (SSC). A clinical trial manager will be appointed. The SMG will meet monthly and oversee day-to-day management and overall conduct and progress of the study including any protocol amendments. The SMG will include all research study team members. The SSC will meet at 6-monthly intervals and will include an independent Chairperson and two other independent expert members who can provide specific advice on methodology, nursing, bronchiolitis and critical care. There will also be representatives from the SMG. The purpose of the SSC will be to provide support and guidance on the conduct of the study and ensure that the study complies with good clinical practice (GCP) principles, relevant regulations and adherence to study protocol. Two parents will be invited to participate in the study management and oversight process. The SSC will report to the funding body.

### Study design

**Fig. 1:**
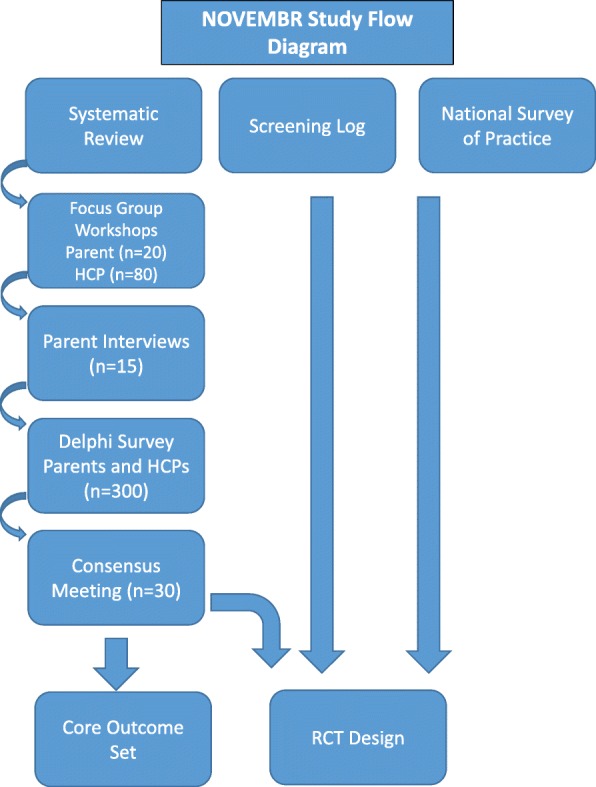
NOVEMBR study flow diagram

This mixed methods study will include a systematic review, focus group workshops (parents/carers and HCPs), telephone/Skype interviews (parents/carers), a Delphi survey (parents/carers and HCPs), a national survey of practice (lead paediatricians) and a consensus meeting (parents/carers and HCPs). These methods will be undertaken to identify and prioritise important outcomes for inclusion in a COS and to explore important issues which will inform the design of a future RCT. In addition, UK hospitals will be contacted and asked to complete a survey to provide an overview of current practice to enable assessment of capability and capacity to run a future clinical trial. Figure 1 illustrates the NOVEMBR study flow diagram.

### Core outcome set (COS) scope and development

The scope of the COS will principally be developed for use in RCTs of interventions (pharmacological or non-pharmacological) for children with a clinical diagnosis of bronchiolitis in a hospital setting. Methods recommended by Core Outcome Measures in Effectiveness Trials (COMET) and COnsensus-based Standards for the selection of health Measurement INstruments (COSMIN) will be used to guide the development of the bronchiolitis COS [48, 51, 52]. A list of outcomes will initially be created from the systematic review and stakeholder consultation (workshops and interviews). Similar outcomes will be collapsed and merged. A conceptual outcomes framework has been developed based on previous exploratory work^1^ (unpublished data) [48, 53]. The outcome domains and sub-domains for the framework will be defined a priori. We will categorise the list of outcomes under relevant domains and sub-domains in the outcome framework prior to inclusion in the Delphi survey. Obtaining consensus for importance for identified outcomes will be an iterative process. A variety of consensus methods will be used within the stakeholder workshops, interviews and Delphi survey, culminating in an end-of-study consensus meeting.

#### Systematic review of outcome measures

The following methods for systematic review of outcome measures have been adapted for use from the mOMEnt study [54, 55].

Studies will be eligible for inclusion if they meet the following inclusion criteria:

1. Study design: RCTs will be included, regardless of specific RCT design.
2. Participants: Infants and children up to 24 months of age with a clinical diagnosis of bronchiolitis in any setting (outpatient, inpatient, paediatric critical care) will be eligible. To accommodate different perspectives on bronchiolitis definition, a pragmatic approach will be used as defined by RCT authors. A priori studies with participants known to have had recurrent wheezing will not be excluded.
3. Interventions: All interventions (pharmacological, medical devices or other) at the patient level will be considered, including but not limited to HFNC, nCPAP, nebulised hypertonic saline, bronchodilators, corticosteroids, surfactant therapy, antibiotics, deoxyribonuclease, steam inhalation or humidified oxygen, heliox, leukotriene inhibitors, epinephrine, glucocorticoids, chest physiotherapy, suctioning, inhaled corticosteroids, oxygen saturation, fluids and nutritional support.

Studies will be excluded if they:

1. Do not recruit exclusively participants with bronchiolitis
2. Include participants diagnosed with bronchiolitis obliterans
3. Evaluate the effectiveness of monoclonal antibody (palivizumab) for the prevention of bronchiolitis
4. Test interventions implemented at the population level
5. Are not published in one of the following languages: Dutch, English, French, German, Italian, Portuguese, Spanish and Turkish.

The following electronic databases will be searched to identify relevant RCTs: OvidSP MEDLINE (1946–2015), Central Register of Clinical Trials (CENTRAL; the Cochrane Library), OvidSP Embase (1974–2015), Scopus (1982–2015). See Additional file 2.

Titles and abstracts will be screened independently by two authors (CvM and RF), and full publication will be obtained for potentially eligible studies. Two researchers (CvM and RF) will independently assess eligibility of the full reports against the inclusion criteria. Disagreements will be resolved through discussion. A data extraction form will be developed and pilot tested for use. Data extracted from eligible studies will include author details; reference; country; setting; number of centres; study duration; population; sample size; study inclusion/exclusion criteria; intervention(s); length of follow-up; and reported outcomes, including any primary/secondary outcomes, definition and when and how they are measured. The data extraction forms will be compared for agreement. Disagreements will be resolved through discussion. If the published data are unclear or unavailable, the study authors will be contacted for further clarification. The quality of describing and reporting the outcomes will be assessed within each study by considering the following questions [54, 55]:

1. Is the primary outcome clearly stated?
2. Is the primary outcome clearly defined so that another researcher would be able to reproduce its measurement?
3. Are the secondary outcomes clearly stated?
4. Are the secondary outcomes clearly defined?
5. Do the authors explain the use of the outcomes they have selected?
6. Are methods used to enhance the quality of outcome measurement (for example, repeated measurement, training) if appropriate?

For this study there will be no statistical synthesis of outcome data of included studies. The methodological quality or risk of bias of the included studies will not be evaluated. The extracted data will be entered into a Microsoft Excel database to aid tabulation and data analysis. For analysis purposes, the data will be initially tabulated so that each study is listed with the outcomes measured. Outcomes will be grouped under appropriate outcome domains by one author (CvM). These groupings will be checked by co-authors. The outcome domains will be determined based on a predefined conceptual framework that will be developed based on previous exploratory work and adjusted as needed following a review of the extracted outcomes by the authors (Footnote 1: unpublished data) [48]. The outcome domains and included outcomes will be reviewed by the SSC to assess suitability of domain name and grouping of outcomes. Within each domain, we will be able to evaluate how many different outcomes have been used to reflect that domain, the frequency of selection for each individual outcome and the times at which they were measured. Tables will be created describing every parameter related to the outcomes used and will refer to which trials reported them. Stratification will be done by intervention and setting in which the trial was conducted, and primary and secondary outcomes will be identified. A narrative synthesis summarising the findings will be undertaken.

#### Focus group workshops and telephone/Skype interviews

Focus group workshops and telephone/Skype interviews with stakeholders (parents/carers and HCPs) will be used to identify outcomes and explore perceptions on the design of a future trial of non-invasive ventilation in children with bronchiolitis. This will include the use of consensus methods to prioritise and obtain agreement on specific aspects of the trial design including primary and secondary outcome measures. Stakeholder acceptability of a future trial will also be determined. Parents/carers and HCPs will be eligible if they meet the following criteria:

1. Parents/carers of a hospitalised child (including ED attenders), aged 0–24 months, with a clinical diagnosis of bronchiolitis defined as per National Institute for Health and Care Excellence (NICE) Bronchiolitis Guidelines (2015) [56].
2. HCPs (doctors, nurses, physiotherapists) who have at least 6 months’ experience in managing children diagnosed with bronchiolitis in the following clinical locations: ED, acute assessment unit, medical ward and critical care unit.

Parents/carers will be excluded from participation if they do not speak English or if their child had died during hospital admission.

A purposive sampling framework will be used to recruit up to 35 parents/carers (workshops (*n* ≤ 20); interviews (*n* ≤ 15)) and approximately 80 HCP stakeholders. To ensure maximum variation, stakeholders will be stratified by specific characteristics. Parents/carers will be stratified by their child’s gender, severity of illness and age, whilst HCPs will be stratified by profession type, grade and clinical area. The sample will be reviewed as recruitment is in progress and will be amended as necessary to avoid over-recruitment of stakeholders with similar characteristics.

A member of the local research team will disseminate written and verbal information about the study to consultant paediatricians and other members of the direct clinical care teams either through email, individual face-to-face meetings or at appropriate meetings. To maximise recruitment, the direct clinical care team will be asked to identify eligible parents/carers either prospectively during their child’s hospital visit/admission or retrospectively for those children who have been recently discharged home. For retrospective recruitment, a letter will be sent to eligible parents/carers via the clinical care team inviting the parent/carer to contact the research team to register interest.

##### Parent/carer focus group workshop

To facilitate engagement with parents/carers, the workshop will be held at a family-friendly venue with facilities to provide a stimulating environment for children who attend with their parents/carers. A crèche facility will be available with qualified nursery staff. All travel expenses will be reimbursed, and parents/carers will be presented with a gift voucher.

Recruitment for the workshop will take place at three study sites in North West England located close to the workshop venue. Parents/carers will be provided with both written and verbal study information. A week prior to the workshop, parents/carers will be sent a copy of the workshop agenda and the NOVEMBR trial participant information sheet to consider.

At the beginning of the workshop, parents/carers will be advised of the study aims and the format of the workshop and will be told that they are free to withdraw at any point without giving reason. Parents/carers will be given opportunity to ask questions. Written informed consent will be obtained by either a member of the study team or research personnel included on the delegation log.

The workshop will be divided into three focus group sessions (outcomes; study design; consent), each lasting up to an hour. The research team will provide an overview of the study and the aims and objectives of the workshop. Patient and Public Involvement (PPI) contributors will be invited to help facilitate the focus groups. A topic guide will be developed for use in the workshop to explore the following: identification and prioritisation of outcomes; deferred consent (including decision-making in the emergency setting); length and content of the participant information sheets; and acceptability of the proposed trial (including identification of potential barriers for participation). Parents/carers’ advice will also be sought on how best to recruit other parents/carers to participate in a future Delphi consensus survey.

The first focus group session will concentrate on outcome identification and prioritisation. A list of outcomes and definitions will be prepared in advance to present to parents/carers. Outcomes will be identified from published bronchiolitis Cochrane systematic reviews and NICE guidance and tabulated. Parents/carers will be asked questions in relation to their experience of having a child with bronchiolitis to identify important outcomes. All outcomes identified by the parents/carers will be recorded on flip-charts. Outcomes identified that are not already on the prepared list will be included on the list for prioritisation. The focus group will discuss each outcome in the prepared list to clarify meaning, and similar identified outcomes will be merged. Using TurningPoint software, parents/carers will be asked to prioritise outcomes using a Likert scale (1, Extremely important to 5, Completely unimportant). Those outcomes which are considered either extremely important or important will be included in a prioritisation exercise in the second focus group.

The second focus group will consider various features of an RCT study design. Parents/carers will be given a demonstration of different methods of providing oxygen therapy and non-invasive respiratory support (HFNC and nCPAP) to children with bronchiolitis using baby mannequins. Two different study design options will be discussed and explored: HFNC compared with standard care and HFNC compared with nCPAP. Parents/carers will be asked what they feel about the different interventions and comparisons, how they would feel if their child was involved in these studies and what outcomes they deem important for each of the two study design options. Following the discussion of the two different study designs, parents/carers will be asked which study design option should be prioritised. The outcomes considered important or extremely important from the previous focus group will be written on Post-it Notes. Using a prioritisation grid (see Additional file 3) designed specifically for this study, parents/carers will be asked to prioritise nine of these outcomes for the preferred study design [57]. Parents/carers will be consulted on what they would consider to be a minimally important difference for the primary outcome measure on which to base a sample size calculation, whenever feasible. For example, if parents/carers prioritised length of hospital stay, we would ask them what they would consider to be an important reduction.

The final focus group in the workshop will explore parents/carers’ perceptions of prospective consent and research without prior consent in relation to the two study designs previously explored [58]. Parents/carers will be provided with a draft participant information sheet for research without prior consent for consideration. Finally, parents/carers will be consulted over what is the best method to use to approach other parents/carers with an invitation to participate in a future Delphi survey.

##### Parent/carer telephone/Skype interviews

Interim findings from the workshop will be used to inform and develop the telephone/Skype interview topic guide as part of an iterative (reflective) approach to the research, although the key topics and structure are likely to be similar to those of the workshop focus group topic guide.

Recruitment to telephone/Skype interviews will take place at all seven study sites. If a parent/carer is recruited prospectively, then written informed consent will be obtained at the site. Parents/carers identified retrospectively who contact the study team will be included on a register with their contact details. Approximately one week prior to the interview, all parents/carers will be sent a copy of the information sheet, consent form, list of outcomes previously developed for use in the parent workshop and copies of the NOVEMBR study (draft) participant information sheet to consider. Parents/carers will be contacted by the research team to ascertain whether they still wish to participate in the interview, to allow them to ask questions and to arrange a convenient date and time for the interview. Parents/carers will be able to select whether they would prefer a telephone or Skype interview.

The researcher will begin the call by explaining the aims of the study and provide further opportunity to ask questions. Parents/carers will be advised that they are free to withdraw from the study at any point without giving reason. They will also be asked for permission to use any data already collected. Data may include age, gender, risk factors, severity of illness and the first part of the postcode of the family home. Parents/carers, irrespective of being recruited prospectively or retrospectively, will be guided through the consent form and verbal consent will be obtained. This verbal consent process will be digitally audio recorded and saved on a separate file from the interview recording.

Interviews will be conducted with up to 15 parents/carers based on similar studies [59] or until data saturation is achieved (i.e. when no more new themes are identified). All interviews will be conversational and participant-centred to ensure that the interview content reflects their own priorities and views on the proposed trial design. It is anticipated that interviews will take no longer than 60 min. To identify important outcomes, parents/carers will be asked about their experience of having a child with bronchiolitis and then be requested to look at the outcome list. The researcher will go through each outcome on the list to check understanding and provide an explanation if required. Parents/carers will be asked to identify which outcomes on the list are important to them. Using a prioritisation grid, parents/carers will be asked to rank the preidentified outcomes in order of importance. Finally, parents/carers will be asked to comment on the draft participant information sheet for a potential future clinical trial.

##### HCP focus group workshops

Recruitment of HCPs will take place at all seven study sites. Information and invitation to participate in the study will be disseminated via health professional organisation global mailing lists or through a brief presentation at appropriate clinical team meetings. Clinical leads will be contacted to suggest an HCP who may fit the inclusion criteria. An HCP may also be approached directly by a member of the research team and invited to participate. In addition, HCPs who participated in two recent National Institute for Health Research (NIHR)-funded bronchiolitis studies (UKCRN 10194 and UKCRN 10755) will be re-approached by email. HCPs will be invited to contact the research team to register an interest to take part in one of three workshops situated at different geographical locations around England. The HCP will be asked to provide the following information: contact details; profession type; grade; clinical location; geographical area. A list of those HCPs who register an interest in taking part in the study will be used to purposively sample by profession, grade, clinical speciality, hospital setting (secondary or tertiary) and geographical location. A member of the research team will contact those HCPs who have been selected to explain key aspects of the study information, including the purpose of the study, what is involved and the risks and benefits of participation. Care will be taken to ensure that HCPs do not feel coerced to participate in the study, and they will be advised that they can withdraw from the study at any point without giving reason. If an HCP withdraws, permission will be sought from the HCP to use any data already collected.

As with the parent/carer workshop, there will be three focus groups (outcomes; study design; consent) within the workshop, each lasting approximately an hour. The research team will use a workshop topic guide, similar to the one used with the parents/carers, to explore identification and prioritisation of outcomes; consent (including decision-making in the emergency setting) [58]; length and content of the participant information sheets; and acceptability of the proposed trial (including identification of potential barriers for participation). Written informed consent will be obtained at the beginning of the workshop from each HCP participant by either a member of the research team or personnel included on the delegation log.

In the first focus group the list of outcomes and definitions prepared for the parent/carer focus group will be provided for use. HCPs will be asked to reflect upon their experience of managing children with bronchiolitis to identify additional outcomes. All identified outcomes will be recorded on flip-charts. The group will discuss each outcome to clarify meaning, and similar outcomes identified from the workshop discussions may be merged. All additional outcomes identified that are not already on the prepared list will be included for prioritisation. Using TurningPoint software, HCPs will be asked to prioritise outcomes using a Likert scale (1, Extremely important, to 5, Completely unimportant). Those outcomes which were considered either extremely important or important will be recorded on Post-it Notes and included in a prioritisation exercise in focus group two.

Two different study design options will be discussed and explored in focus group two: HFNC compared with standard care and HFNC compared with nCPAP. HCPs will be asked to consider and discuss topic areas related to the design of the clinical trial, such as study interventions and acceptability of a clinical trial of non-invasive ventilation. HCPs will be asked which of the two trial designs should be prioritised. Using a prioritisation grid [57] and the outcomes recorded on Post-it Notes from focus group one; HCPs will be asked to prioritise nine outcomes, including a primary outcome. Similar to the parent/carer focus group, HCPs will be consulted on what should be considered as the minimally important difference for the primary outcome. In the third focus group HCPs will be asked to consider the use of prospective consent and research without prior consent [58] in relation to the two study designs previously discussed. HCPs will be provided with a draft participant information sheet for research without prior consent for consideration.

##### Qualitative data analysis and storage

All focus groups and interviews will be audio recorded and transcribed verbatim by an external transcription company. Transcripts will be pseudo-anonymised. Audio files will be deleted once transcribed; with the exception of the audio recorded consent for parent/carer interviews. All study data will be stored according to data protection requirements and local data governance policies. Identifiable participant data will be kept in a file separate from the anonymised participant data on a secure server on a password protected NHS computer. All identifying details such as names, dates of births and hospitals will be removed and replaced with pseudonyms. All participants will be identified with a unique identifier. All recordings, transcripts and documents will be coded with the unique participant identifier to ensure anonymity. Electronic versions of the transcripts will be stored on the main server of a password protected computer.

Digital audio recordings of focus groups and interviews will be transcribed verbatim. Other data sources derived from focus groups and interviews will include lists of words and phrases written down by the participants and words and phrases recorded on flip charts; field notes made by the researchers will also be analysed. Transcripts and all other data sources will be examined iteratively several times over the course of the analysis. Synthesis of all types of data sources will be undertaken using a constant comparative method [60]. Initial examination of the data will aim to provide an overview of the data with general impressions recorded, including key ideas, themes and concepts arising from the content. A descriptive thematic analysis will then be undertaken [61]. This process will involve manually coding the raw data, then collapsing the coded data under broader themes. Codes and themes will be inductively derived from the data. As new codes emerge, they will be applied iteratively to the whole data set in subsequent examinations of the data sources until no new codes or themes are identified. A descriptive account will be produced for each theme. Finally, outcomes identified from transcripts and other data sources will be grouped under appropriate domains and sub-domains using the predefined conceptual bronchiolitis conceptual framework. QRS NVivo (version 10) software will be used to support the coding and synthesis of data. Field notes will be used to support the analysis through describing the environmental geography, participant interaction, group dynamics, behaviour and non-verbal communication. Field notes will also enable the researcher to reflect on the focus groups and interviews and record any meaningful thoughts and insights.

#### Delphi survey to prioritise outcomes to include in a COS

In the third and final stage, we will undertake a Delphi survey to reach consensus over which outcome measures to include in a COS for trials in the management of children with bronchiolitis. We will adopt this approach to ensure the anonymous opinions of important stakeholders (parents/carers and HCPs) are obtained in a way that gives equal influence to all who participate, and avoids an individual participant being overtly influenced by the opinions of other participants [62, 63].

We will review all outcomes identified from the systematic review and stakeholder consultation (workshops and interviews) for inclusion in the Delphi survey. Outcomes considered similar (for example, those that measure the same phenomenon) will be collapsed and merged. Each outcome will be categorised under the relevant domains and sub-domains in the outcomes framework defined a priori, as previously stated.

Stakeholders who meet the following eligibility criteria will be invited to complete the Delphi survey:

1. Parents/carers of a child hospitalised between 2016 and 2018 (including ED attenders), aged 0–24 months, with a clinical diagnosis of bronchiolitis defined as per NICE Bronchiolitis Guidelines [56]
2. HCPs (nurses, doctors, physiotherapists) who have at least 6- months experience in managing children diagnosed with bronchiolitis.

We will also invite eligible stakeholders who have been involved with earlier phases of the study to participate. Parents/carers who do not speak English or whose child has died during hospital admission will be excluded.

##### Sample and setting

There is currently no standard method to determine sample size for the Delphi survey. A pragmatic decision to recruit up to 40 parents and up to 300 HCPs was taken based on other studies [64, 65]. Efforts will be taken to maximise the response rate across centres and stakeholder groups.

Eligible parents/carers who were approached to participate in workshops or interviews will be asked to complete a permission to contact form. This form will include a box to be ticked to indicate interest in participation in the Delphi survey. In addition, we will invite several UK hospitals to become participant identification centres (PICs). A member of the research team at each PIC will identify eligible parents and provide them with the Delphi information sheet. The information sheet outlines the Delphi survey process and provides instructions on how to contact a member of the study team for more information. PICs will also be asked to display posters advertising the Delphi survey in relevant clinical areas. We intend to make links with a number of general or respiratory-specific patient advocacy groups in order to circulate the poster and the contact details for the research team.

To identify eligible HCPs, the study team will send an email advertising the Delphi survey to professional organisations for them to distribute to members via global email address lists or associated social media sites. Professional organisations will at least include the following: NIHR Clinical Research Network: Children; General and Adolescent Paediatric Research Collaborative - UK and Ireland (GAPR-UKI); Acute Paediatric Emergency Medicine (APEM); Royal College of Nursing Children’s and Young Persons Forum. We will ask PICs to circulate Delphi survey information to the relevant HCPs within their organisation. In addition, clinicians who complete the national survey of current practice will be asked to provide their email address if they wish to be contacted regarding participation in the Delphi survey.

All participants will be invited to pass on details of the study to any of their own contacts who meet eligibility requirements. Potential participants will be given details of how to register when they contact the study team. Screening questions will be included on the Delphi registration page for potential participants to complete. If they do not meet these criteria, then they will be unable to complete the registration process. Eligible participants will be included on a register and allocated a unique identifier. We will use the unique identifier to anonymise and store data and to track attrition rates between rounds. The following information will be collected for each stakeholder group:

1. Parents/carers: When the respondent’s child was hospitalised with a diagnosis of bronchiolitis
2. HCPs: Respondent’s clinical role; whether respondent works in a secondary care, tertiary care or ‘other’ setting; name of respondent’s employing organisation; number of years post-professional registration; grade.

A statement will be included on the Delphi registration page highlighting that completion of the questionnaire will be regarded as consent.

##### Delphi survey methods

We will upload outcomes identified from the systematic review and stakeholder consultation onto the web-based Delphi system (Delphi Manager Application version 1.1). This system will facilitate management of the Delphi survey. A hyperlink to access the survey will be embedded in an email along with the study information sheet. The information sheet will provide participants information on the NOVEMBR study and the Delphi survey, notify participants that their participation is voluntary and stress the importance of completing all Delphi survey rounds. Furthermore, we will inform participants that if they complete all Delphi survey rounds they will be entered into a prize draw to win an iPad and that HCPs will receive a certificate of completion. The Delphi survey will be pilot tested prior to distribution to determine any technical issues with the software, clarity of wording, time taken to complete the survey and ease of use, and the survey will be refined as necessary. We will ask participants to complete each round of the Delphi survey within four weeks following receipt of the email. Participants will be reminded of this at the start of each survey. A reminder email will be sent out at the end of the first week to prompt completion of the survey. Withdrawals will be classed as those participants who contact the NOVEMBR study team directly or write a comment in the survey comments box to indicate they do not wish to participate in any more Delphi survey rounds.

Delphi survey round one will contain:

1. A list of outcomes to be scored, ordered alphabetically by domains. The list of outcomes will include the option to display a more detailed plain language description. The text will be reviewed by the study team and parent representative.
2. An option for a participant to add any additional outcomes and to provide a score for each outcome added.

At the beginning of the survey, participants will be asked the following key question:

*What outcomes are most important in the management of children with bronchiolitis?*

Participants will be asked to score each of the outcomes listed using the Grading of Recommendations, Assessment, Development and Evaluation (GRADE) scale of 1–9 [66]. In the Delphi exercise the scale will be presented in the format 1–9 with 1–3 labelled as 'not important', 4–6 labelled as 'important but not critical', 7–9 labelled as 'critical' and the choice 'unable to score' [66]. Participants will be provided with an option to add additional outcomes that they think are relevant together with a score for each outcome added. Outcomes will be listed alphabetically to avoid potential weighting caused by the order in which they are displayed. Additional outcomes listed by participants will be reviewed and coded by members of the study team to ensure they represent new outcomes. The SMG will be consulted if there is uncertainty. For each outcome, the number of participants who have scored the outcome and the distribution of scores (as a percentage of those who have scored each outcome) will be summarised based on stakeholder group. All outcomes will be carried forward to round two.

The number of participants in each stakeholder group who respond to round one will be assessed following round one closure. Results will be presented as:

- Total number of registrations
- Breakdown of respondents who have completed the survey and their inclusion in the initial email invitation
- Total number of respondents who completed the round
- Total number of respondents in each stakeholder group
- Percentage of respondents compared to potential respondents as identified from the information provided by clinical leads
- Percentage of respondents from other sources (not included in original email invitation)

If a low number of responders (less than 10) is observed for one or more stakeholder groups, the Delphi protocol for future rounds will be reviewed and revised. Where there is only one stakeholder group with a small number of respondents (potentially due to the sample available from clinical teams), then consideration will be given to grouping with another stakeholder group; e.g. physiotherapists may be grouped along with nursing staff. This will be done in consultation with the SMG to ensure appropriateness of grouping. The proposed approach assumes sufficient numbers of stakeholders from each group who respond. Continuation to round two will be considered based on the response to round one. Those who have not taken part in round one and not provided a score will not be invited to participate in round two.

In round two, participants will be shown their own scores for each outcome as well as the scores given by each stakeholder group using a bar chart displaying the distribution of scores. Participants will then be asked to re-score all outcomes and state whether they should be included in a COS. Participants will be provided with the option to explain any significant score changes. Round two will be presented online. The total number of participants invited to take part in round two will be recorded. For each outcome, the number of participants who have scored the outcome and the distribution of scores will be summarised together with the number of participants who have scored the outcome in all rounds. Results of the stakeholder group response will be compared to the whole group response, and the percentage agreement used to determine the structure and focus of the final consensus meeting. Each outcome will be classified as either ‘consensus in’, ‘consensus out’ or ‘no consensus’ as described in Table 1 [66].

**Table 1:** Consensus classification for all stakeholder groups

Consensus classification	Description	Definition
Consensus in	Consensus that outcome should be included in the core outcome set	70% or more participants scoring as 7–9 AND < 15% participants scoring as 1–3 in each group
Consensus out	Consensus that outcome should not be included in the core outcomes set	< = 50% of participants scoring as 7-9 in each group
No consensus	Uncertainty about importance of outcome	Anything else

For consensus to have been reached that an outcome should be included in the COS, there must be agreement by a clear majority on the importance of the outcome with only a small minority considering it to be not important at all. For consensus to have been reached that an outcome should not be in the COS, there must be agreement by a clear majority on the lack of importance of the outcome with only a small minority considering it to be important. Whilst the choice of thresholds is inevitably somewhat subjective, the definition of consensus a priori should reduce the chance of consensus being defined post hoc in such a way as to bias the results towards the beliefs of the research team [48]. Once the final analysis of the online Delphi survey has been conducted, the results will be summarised, a report will be written by the study researcher and recommendations for future research will be made based on the findings of the study.

#### National survey of practice

To investigate current practice in the management of children with a clinical diagnosis of bronchiolitis, a national survey of practice will be developed. The survey will be pilot tested and circulated by the Liverpool Clinical Trials Research Centre (CTRC) to UK hospitals and paediatric research groups. The survey will be developed using SurveyMonkey® software. Lead paediatricians for each NHS trust will be sent an email inviting them to complete the survey, which will be accessed via a hyperlink embedded into the email. The survey will cover topics such as current treatment, method of delivering treatment, available facilities and staffing levels, level of staff training and whether the survey participants believe it would be feasible to run a randomised control trial of non-invasive ventilation in children with bronchiolitis at their hospital. Furthermore, there will be a request within the survey for lead paediatricians to forward local bronchiolitis guidance to establish variation in practice.

In addition to the survey, screening logs will be completed, for six weeks, over two bronchiolitis seasons at five sites (until March 2018). The purpose of the screening logs is to collect admission data, which will include age upon admission, length of patient stay, referral route, risk factors, reason for admission, treatment, oxygen therapy and patient outcome. The screening logs will provide further information on current management of children with bronchiolitis and the proportion of children who require non-invasive ventilation. Once the survey and screening logs have been completed, they will be returned to the CTRC, where the responses will be collated and analysed by the study statistician.

#### Consensus meeting

The final phase of the study will be a face-to-face consensus meeting. All participants involved with either stakeholder consultation and/or Delphi will be invited to attend. The final format of the consensus meeting will be determined following review of the experiences of previous similar projects [67]. This will include deciding on the Chair and attendees, recruitment and consent, list of outcomes to be presented for discussion and the consensus methods. Results from the systematic review, stakeholder consultation, Delphi survey and national survey of practice will be presented. There will then be a final discussion and vote on the trial design and bronchiolitis COS.

## Discussion

This feasibility study protocol describes how we plan to design a future clinical trial to investigate the efficacy of non-invasive ventilatory support and the development of a COS for children with bronchiolitis. The current published literature demonstrates a real need for further well-designed trials that include outcomes relevant to important stakeholder groups. Furthermore, a bronchiolitis COS would greatly improve measurement and reporting of outcomes in future research. Therefore, this study will contribute to the burgeoning evidence base in this area and, one hopes, improve the future healthcare of these children.

### Proposed clinical trial

This feasibility study will enable us to make important decisions regarding any future clinical trial such as whether a trial is necessary, which interventions to prioritise and which primary and secondary outcomes to use. Furthermore, the screening log and national survey of practice will help identify issues with capability and capacity, such as variation in practice; availability of equipment; and staffing issues and training requirements. If a trial is thought to be necessary, then the final output of this study will be to develop a protocol for such a trial with a view to seeking funding in the UK.

### Core outcome set development

There is no COS for children with bronchiolitis. The methodology we have described for COS development adheres to the recently published Core Outcome Set-STAndards for Development (COS-STAD) recommendations, which describe three key domains important in the development of any COS: scope, stakeholder involvement and consensus process [51, 68].

The scope of the COS includes children up to 24 months old with a clinical diagnosis of bronchiolitis in a hospital setting (standards 1, 2, 3 and 4) [51]. Moreover, we intend to incorporate both pharmacological and non-pharmacological interventions. There are limitations concerning the scope. Firstly, it is restricted to children residing in the UK in terms of bronchiolitis definition and setting. This may preclude the uptake of the COS internationally. Further evaluation in countries outside the UK may be necessary to mitigate this limitation. Secondly, the COS is limited to a hospital setting. A large proportion of children with bronchiolitis are currently managed in the community. It may be necessary for future studies to address this shortcoming. The presupposition is that a bronchiolitis COS will be primarily used for future clinical research. However, other potential applications for the bronchiolitis COS could include quality improvements in clinical practice and guideline development.

Notably, we will involve and consult important stakeholders throughout this study. This important contribution will help ensure that future research outputs, in terms of the clinical trial and COS, will be both acceptable and relevant to all major stakeholder groups. The inclusion of HCPs and parents/carers meets the COS-STAD recommendations (standards 6 and 7) for developing a COS [51]. A limitation with respect to stakeholder involvement is the lack of engagement with industry partners as participants. These stakeholders were not considered for inclusion because, at the time the protocol for this study was developed, there were no specific treatments for either bronchiolitis or the viruses that cause bronchiolitis. This has recently changed; antiviral medications are undergoing early phase clinical trials [69, 70].

The consensus process for identifying, including and excluding outcomes has been clearly described a priori in accordance with COS-STAD recommendations (standards 8, 9, 10 and 11) [51]. Stakeholders (parents/carers and HCPs) will be fully involved in this process through focus groups, interviews, a Delphi survey and a consensus meeting (standard 8) [51]. We are using the GRADE scale to score the list of outcomes for importance [66], and consensus will be defined using the definition developed for the mOMEnt study [54] (standards 9 and 10) [51]. HCP and parent representatives will be on the SMG to review all outcomes for ambiguity of language (standard 11).

Finally, future research for the definitive bronchiolitis COS will include exploring how best to measure each of the outcomes included.

## 3.3. Trial status

At the time of manuscript submission, the NOVEMBR feasibility study was still open to recruitment. The NOVEMBR study opened for recruitment on 12/2/2016 (Protocol v1.0 Date: 19.10.15), and recruitment was completed by 14/6/2018.

## Additional files

Additional file 1: NOVEMBR aims and objectives. (PPTX 66 kb)

Additional file 2: NOVEMBR search strategy. (PDF 68 kb)

Additional file 3: NOVEMBR prioritisation grid. (PDF 103 kb)

## Abbreviations

ED: Emergency department
HCP: Healthcare professional
HFNC: High-flow nasal cannula
nCPAP: Nasal continuous positive airway pressure
NHS: National Health Service
NIV: Non-invasive ventilation
PIC: Participant identification centre
PICU: Paediatric intensive care unit
RCT: Randomised controlled trial
REC: Research Ethics Committee
SMG: Study management group
SSC: Study Steering Committee

## Footnotes

1 Fernandes RM. Interventions and Outcomes in Bronchiolitis Clinical Trials. Lisboa, Portugal: UNIVERSIDADE De LISBOA 2015 (unpublished data). http://repositorio.ul.pt/handle/10451/22714?locale=en
