# Supplementary material for: Non-invasive ventilation for the management of children with bronchiolitis (NOVEMBR): a feasibility study and core outcome set development protocol
Source: Trials. 2018 Nov 14;19:627. doi: 10.1186/s13063-018-2969-9 (PMC6236891; doi:10.1186/s13063-018-2969-9)
Supplement: Supplementary file 1 — NOVEMBR aims and objectives. (PPTX 66 kb) [file 13063_2018_2969_MOESM1_ESM.pptx]

## Slide 1
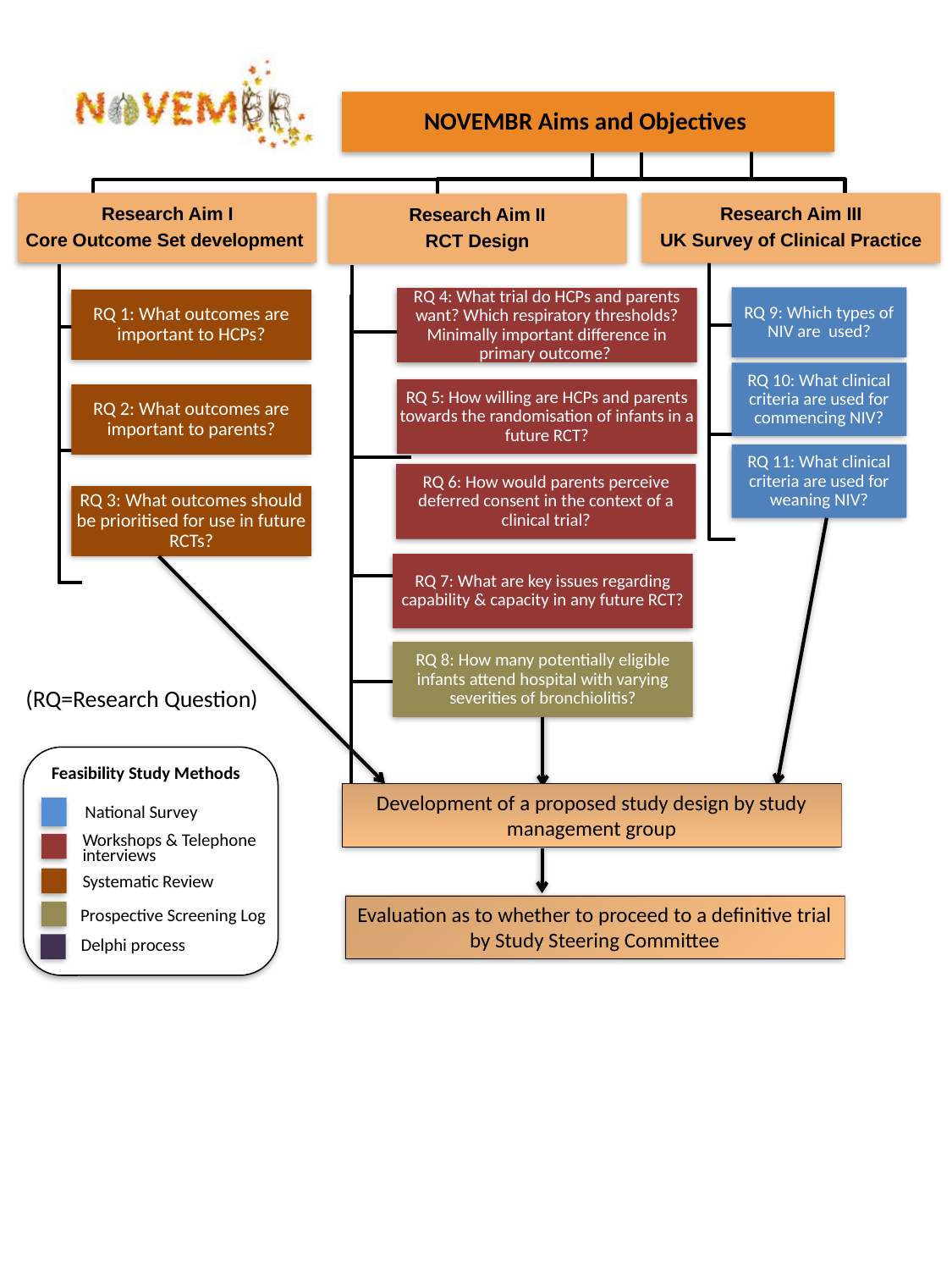

NOVEMBR Aims and Objectives
Research Aim I
Core Outcome Set development
Research Aim III
UK Survey of Clinical Practice
RQ 9: Which types of NIV are used?
RQ 10: What clinical criteria are used for commencing NIV?
RQ 11: What clinical criteria are used for weaning NIV?
Research Aim II
RCT Design
RQ 4: What trial do HCPs and parents want? Which respiratory thresholds? Minimally important difference in primary outcome?
RQ 1: What outcomes are important to HCPs?
RQ 5: How willing are HCPs and parents towards the randomisation of infants in a future RCT?
RQ 2: What outcomes are important to parents?
RQ 6: How would parents perceive deferred consent in the context of a clinical trial?
RQ 3: What outcomes should be prioritised for use in future RCTs?
RQ 7: What are key issues regarding capability & capacity in any future RCT?
RQ 8: How many potentially eligible infants attend hospital with varying severities of bronchiolitis?
Development of a proposed study design by study management group
National Survey
Workshops & Telephone interviews
Systematic Review
Prospective Screening Log
Delphi process
Evaluation as to whether to proceed to a definitive trial by Study Steering Committee
(RQ=Research Question)
Feasibility Study Methods
