# Supplementary material for: Non-invasive ventilation for the management of children with bronchiolitis (NOVEMBR): a feasibility study and core outcome set development protocol
Source: Trials. 2018 Nov 14;19:627. doi: 10.1186/s13063-018-2969-9 (PMC6236891; doi:10.1186/s13063-018-2969-9)
Supplement: Supplementary file 2 — NOVEMBR search strategy. (PDF 68 kb) [file 13063_2018_2969_MOESM2_ESM.pdf]

Database: OvidSP Medline <1946 to November 2015>

MeSH and text words for Bronchiolitis and Post-Bronchiolitis:
1. exp BRONCHIOLITIS/
2. (bronchiolitis or wheez*).mp.
3. exp Respiratory Syncytial Viruses/ or exp Respiratory Syncytial Virus Infections/
4. Respiratory Syncytial Virus$.mp.
5. (post adj3 (bronchiolit* or RSV or "respiratory syncytial")).mp.
6. (recurrent adj2 (cough* or wheez* or bronchiolitis)).mp.

7. or/1-6 [MeSH and keyword terms related bronchiolitis + post bronchiolitis]

Mesh and text words for Infants:
8. exp Infant/
9. (Infant* or infancy or Newborn* or Baby* or Babies or Neonat* or Preterm* or Prematur* or Postmatur*).mp.
10. exp CHILD/
11. (child* or preschool* or toddler* or kid? or kindergar* or boy? or girl?).ti,ab,jw,nw.

12. or/8-11 [combination of infant MeSH and keyword terms]

Study Design Filter for Randomized Controlled Trials:
13. randomized controlled trial.pt.
14. controlled clinical trial.pt.
15. randomized.ab.
16. placebo.ab.
17. clinical trials as topic.sh.
18. randomly.ab.
19. trial.ti.
20. or/11-17
21. exp animals/ not humans.sh.

22. 20 not 21 [Cochrane RCT filter for Medline; max sensitivity/specificity]

21. and/7,10,20 [combination of terms for bronchiolitis, infants, RCTs]

Database: OvidSP Embase <1980 to 2015>

Index and text words for Bronchiolitis and Post-Bronchiolitis:
1. exp BRONCHIOLITIS/
2. (bronchiolitis or wheez*).mp.
3. exp Respiratory Syncytial Pneumovirus/
4. Respiratory Syncytial Virus$.mp.
5. (post adj3 (bronchiolit* or RSV or "respiratory syncytial")).mp.
6. (recurrent adj2 (cough* or wheez* or bronchiolitis)).mp.

7. or/1-6 [bronchiolitis + post bronchiolitis terms]

Index and text words for Infants:
8. exp Infant/
9. (Infant* or infancy or Newborn* or Baby* or Babies or Neonat* or Preterm* or Prematur* or Postmatur*).mp.
10. exp CHILD/
11. (child* or preschool* or toddler* or kid? or kindergar* or boy? or girl?).ti,ab,jx.

12. or/8-11

Study Design Filter for Randomized Controlled Trials:
13. random*.tw.
14. placebo*.mp.
15. double-blind*.tw.

16. or/13-15 [Embase RCT filter, J Med Libr Assoc 2006;94(1):41-47]

17. and/7,12,16 [combination of bronchiolitis, infant and RCT terms]

16. remove duplicates from 31

Database: CENTRALvia Cochrane Library

Index and text words for Bronchiolitis and Post-Bronchiolitis:
1. exp BRONCHIOLITIS/
2. (bronchiolitis or wheez*).mp.
3. exp Respiratory Syncytial Viruses/ or exp exp Respiratory Syncytial Virus Infections/
4. Respiratory Syncytial Virus$.mp.
5. (post adj3 (bronchiolit* or RSV or "respiratory syncytial")).mp.
6. (recurrent adj2 (cough* or wheez* or bronchiolitis)).mp.

7. or/1-6

Index and text words for Infants:
8. exp Infant/
9. (Infant* or infancy or Newborn* or Baby* or Babies or Neonat* or Preterm* or Prematur* or Postmatur*).mp.
10. exp Child/
11. (preschool* or toddler* or kid? or kindergar* or boy? or girl?)

12. or/8-11

13. and/7,12 [combination of bronchiolitis terms and infant terms]

14. limit 13 to medline records

15. remove duplicates from 3

Database: Scopus via SciVal

((TITLE(bronchiolitis OR wheez*)) AND (TITLE-ABS-KEY("Clinical Trial" OR "Clinical Trials" OR "Randomized Controlled Trial*" OR "Random Allocation" OR "double-blind method" OR "single-blind method" OR placebos OR research design OR comparative study OR evaluation studies OR follow-up studies OR prospective)) AND (TITLE-ABS-KEY(infan* OR newborn* OR neonat* OR baby OR babies OR Child* OR preschool* or toddler* or kid? or kindergar* or boy? or girl?)))
