# Supplementary figures and images for: Non-invasive ventilation for the management of children with bronchiolitis (NOVEMBR): a feasibility study and core outcome set development protocol
Source: Trials. 2018 Nov 14;19:627. doi: 10.1186/s13063-018-2969-9 (PMC6236891; doi:10.1186/s13063-018-2969-9)

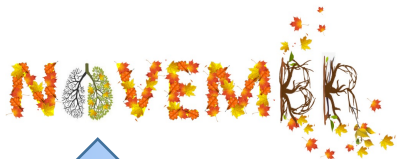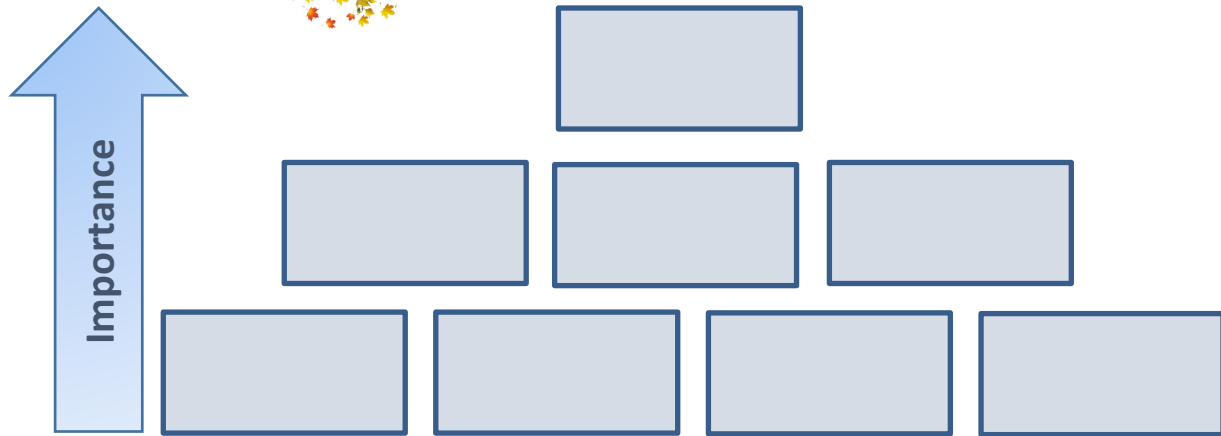

Participants are invited to sort and rank outcomes in order of importance.

Supplement: Supplementary file 3 — NOVEMBR prioritisation grid. (PDF 103 kb) [file 13063_2018_2969_MOESM3_ESM.pdf]
